# "The Hospital" Nursing Mirror

**Published:** 1899-10-21

**Authors:** 


### "The Hospital" Nursing Mirror.

Being the Nursing Section of "The Hospital."

[Contributions for this Section of "The Hospital" should be addressed to the Editor, The Hospital, 28 & 29, Southampton Street, Strand, London, W.C., and should have the word "Nursing" plainly written in left-hand top corner of the envelope.]

### Notes on News from the Nursing World.

### NURSES AND THE WAR.

There is an impression in some quarters that the Central British Red Cross Committee entertain applications from nurses who desire to proceed to South Africa. This is a misapprehension. Major Macpherson, the hon. secretary of the committee, writes us that he has "no information in regard to nurses, as the Army Nursing Reserve is supplying all requirements," and he adds that "the chairman of the committee is keeping the public fully informed as to what aid the committee is affording." With respect to the Army Nursing Reserve, we are informed that 29 members have been called up for home duty, and that 11 have received orders to prepare to sail.

### NURSING THE WOUNDED AT MAFEKING.

One of the pleasing, though not by any means unexpected, features of the war in the Transvaal is that the Sisters of the Convent, though granted permission to leave Mafeking by the Roman Catholic Bishop, have all elected to stay and nurse the wounded. A number of ladies have also volunteered to remain and act as nurses. These brave and self-denying women will probably have plenty of work in the houses which have been converted into hospitals. However desirable it may be that trained nurses should be available, the help of the Sisters and other ladies will, in the circumstances, be immensely appreciated

### NURSING IN THE TRANSVAAL.

Although we do not accept responsibility for the statements about nursing in Johannesburg which are made by a correspondent, they are entitled to attention. Just now, of course, no nurse would propose to go to the Transvaal on her own account, but when the war is over, the pestilence, which usually follows in its train, may devastate a city evidently badly protected against it. The disadvantages of nursing, whether in a hospital or among private patients in Johannesburg, should be carefully weighed before any English nurse thinks of trying her fortunes there.

### THE ROYAL CONVALESCENT HOME AT BRISTOL.

We are able, through the courtesy of the matron, to state that the Royal Convalescent Home at Durdham Down, Bristol, which will be opened by the Queen on November 15th, will contain 80 beds for convalescent patients from the various local institutions; and that the staff will consist of the matron, two trained nurses, and two or three probationers. There will also be a non-resident medical officer. It is hoped that the home will be ready for the reception of patients soon after the opening ceremony.

### THE PAY OF PLAGUE NURSES.

There is not, apparently, the same eagerness to nurse the plague this year as there has been in times past. Nor is this extraordinary, for the terms offered do not equal the earnings of a well-trained competent nurse at home. Many of those who went out two years ago have either returned, or are on the point of returning. It has been found that whilst no one has actually been out of pocket, few, if any, even of the most economical have been able to add to their savings. One of the expenses that rankles in the minds of those on whom it falls is the income-tax. For the Government to pay a nurse nominally 175 rupees a month with one hand, and then to claim five rupees of it back as income-tax seems hard, although this is the custom in other branches of Government employ. Out of the 175 rupees the nurse has to provide quarters, service, and keep in many instances, these expenses varying at every station. In addition to the necessities of life, as understood in England, the nurses find that what is a luxury at home is a necessity in hot climates. Carriage hire in India is a heavy pull on a limited purse, but it is not so expensive as the exhaustion entailed by walking in the heat. The unfortunate recurrence of the plague and the consequent demand for trained nurses may prompt the Indian authorities to improve the conditions offered.

### LORD DUFFERIN ON AMATEUR NURSES.

In a recent speech at Bangor the Marquis of Dufferin made some amusing comments on the solecisms which it is possible to commit in the sick chamber by ladies who essay the part of amateur nurses. "They irritate us by sitting upon our bed. When they wash our faces they spill the water over the bedclothes. When they dry our faces they rub us with a towel that is more like a gravel walk than anything else; and sometimes they administer the wrong bottle and cause very serious catastrophes." Amid much laughter, Lord Dufferin declared that if he himself fell ill he would sooner appeal to the professional knowledge and skill of his daughter, who is qualifying herself as a nurse in the London Hospital, than "to the amateurish efforts of his zealous but incompetent wife."

### THE IMPROVEMENTS AT KING'S COLLEGE HOSPITAL.

On Wednesday a number of visitors, invited by Viscount Dillon and the Committee of Management, inspected the important improvements which have been made in the interior of King's College Hospital. It will be recollected that last year the Visiting Committee of the Prince of Wales's Fund called attention to the state of the floors, which was very deplorable; and, in order to encourage the Hospital Committee to refloor the wards, they made a special grant of £475 for the purpose. The work has now been completed in an admirable manner, and the wards have also been lighted throughout with electricity. There is no doubt that the changes will be appreciated both by the patients and the nurses. The old floors constantly needed washing, and the wards were very insufficiently lighted with gas; whereas the new floors will always be clean and dry, and, with the electric light, the patients will be able to see to read during the long, dark, winter evenings. The cost of the improvements has been about £3,000, of which £800 has been contributed. The public, we hope, will not be slow to make up the deficit of £2,200, for the expenditure in the interests of the charity, which is free to the sick poor, was absolutely needed.

### ROYAL NATIONAL PENSION FUND FOR NURSES.

We are asked to request those nurses who are entitled to certificates, but were unable to attend at the last Marlborough House Presentation, and who have not already applied for them, to do so without delay. In each case the application should be accompanied by a penny stamp, and should be addressed to the Secretary, 28, Finsbury Pavement, E.C.

### NURSING IN FEVER HOSPITALS.

The brisk correspondence which is being pursued elsewhere in our pages is distinctly interesting, though some of the nurses who write to us, as, for example, "Credenda," are decidedly too sweeping in their assertions. We notice that "H. Green," alluding this week to the hospitals under the Metropolitan Asylums Board, says "they are supported by the public, principally the working class." But though they are undoubtedly used principally by the working class they are supported entirely out of the rates. Thousands of the working class who are treated within their walls never directly contribute a farthing to the rates. Last week some useful details were given respecting the practice pursued at a fever hospital in reference to the admission of nurses. The hospital in question is the City Hospital, Edinburgh, and the fact that Miss Sandford, the lady superintendent, has more applications for probationerships than she can entertain, indicates that the training afforded is widely known to be valuable.

### THE NURSES' CO-OPERATION.

The Nurses' Co-operation has been doing exceedingly well this year. Since Christmas the demand for nurses has been increasing, and numbers of applications have been refused every month. It may be remembered that the Red Cross Committee sent out two nurses attached to the staff of this society to the Soudan. Two of the nurses sent to Central Africa this year are also members. Three of the members are in India plague nursing, one is superintendent sister at Poonah, and another is to come home shortly. The staff has several members of the Army Nursing Service Reserve on its list. These, as already intimated, are expected to hold themselves in readiness to take duty in the home military hospitals in the event of the regular army sisters being sent to the seat of war.

### THE NURSES AT THE KENT AND CANTERBURY HOSPITAL,

Complaints among the nurses of the Kent and Canterbury Hospital in reference to the food and other matters have been rife for some time, and lately a petition for "better diet and kinder treatment" was sent to the committee by the sisters and nurses.

### PENSIONS FOR THE NURSES AT THE SURREY COUNTY HOSPITAL.

At the meeting of the governors of the Royal Surrey County Hospital last week, Mr. H. Powell moved a resolution in favour of the adoption of a general pension scheme for matrons and nurses, and approving the scheme for federation with the Royal National Pension Fund, which had been carefully considered by the committee. Mr. Eyre seconded, and the motion was unanimously carried. The essence of the scheme is that every nurse engaged at the hospital will have the option of taking out a policy for a a certain amount to be paid her at the age of 55. If she accepts that option the hospital will pay half the premiums and the nurse the other half. The matron is to retire at 55, at a nominal pension of £40 a year, of which she pays half and the hospital half, the head nurse £20, and the assistant nurses £15.

### THE QUESTION OF UNIFORMS IN AMERICA.

In the report of the second annual convention of trained nurses of the United States and Canada, which has been courteously forwarded to us, there is an interesting little paper by Miss Harriet Fullmer, superintendent of the Chicago Visiting Nurse Association, on "Uniforms." The discussion which followed showed a marked feeling against wearing outdoor uniforms. For example, one speaker said, "I have been doing private nursing, and I dislike to wear my uniform in the street. I think I bring back germs to my patients." Another complained, "It calls out many disagreeable remarks in public places"; while a third urged that "One trouble is that it has been taken up by women of ill-repute, who have obtained admittance in its garb where otherwise they could not go." A curious argument was used on the other side by a nurse who considered a distinctive dress a good thing. In support of her opinion she remarked, "I was in the South with a patient, and wore a white dress and a sailor hat, and I was mistaken for a lady. A lady told me that she would never have dreamed that I was a trained nurse." The same speaker subsequently suggested that if the dress was made ugly no one who was not entitled would want to wear it. We may correct an erroneous impression that was not corrected at the meeting. "I think no one," said Mrs. Hawley, "can wear a nurse's dress falsely in England without being brought before a magistrate." There is no law on this side of the Atlantic which makes it a penal offence to masquerade as a nurse.

### THE FRICTION AT LOWESTOFT HOSPITAL.

As a special committee of the Governors of Lowestoft Hospital will at the close of this week issue their report upon the difficulties between the matron and Dr. Walker, we forbear from commenting upon the matter at issue. But the proceedings at the annual meeting point to the conclusion that some change in the *personnel* at the hospital is absolutely necessary. The vital point, as Sir Savile Crossley signified in his speech on that occasion, is "that the hospital should not suffer any permanent harm"; but with the officials at loggerheads it is impossible to prevent an institution from losing ground.

### A BITTER CRY FROM THE EAST-END OF LONDON.

Here is a cry from a big Poplar parish: "Then we want a parish nurse. The one we get from the East London Fund has to look after two parishes—17,000 people. Moreover, we want a nurse to do all the dressings (*e.g.*, of those cases which are seen by a doctor who gives his services weekly). Besides, the nurse must go every morning into the schools, for there are eyes and ears there, to say nothing of toes and other limbs, which mean future uselessness, but which could now be summarily dealt with and cured." If the 17,000 people in these parishes are not able to pay for a nurse, perhaps some generous person may be willing to provide the salary. But a trifling contribution from a modest proportion of the 17,000 would suffice, and an earnest attempt should be made to obtain the money needed from those who require the nursing.

### THE IMPORTANCE OF A BUTTON.

A somewhat unprofitable controversy has come to a satisfactory close at Liverpool. Miss Shaw, a young lady from Ireland, who desired to enter the workhouse infirmary at Brownlow Hill as probationer, complained that Miss Stewart, the superintendent, in an interview on the subject made some disparaging remarks about Irish girls. Subsequently, Miss Stewart candidly admitted that she said untidiness was a failing of Irish girls, but denied that she charged them with being "dirty." The absence of a button on the jacket of Miss Shaw seems to have suggested the charge of untidiness; but all aspects of the dispute were ventilated at a full meeting of the Liverpool Workhouse Committee last week. In justice to Miss Stewart, it should be stated that Miss Thorburn, one of the committee, mentioned that the superintendent had told her that some of the very best nurses she had were Irish girls; and she also gave instances of the devotion and affection of Irish nurses to their patients and to Miss Stewart, as well as of Miss Stewart's fondness for her Irish nurses. The committee, therefore, did well to reject the hypothesis that Miss Stewart has any prejudice against Irish nurses merely because she used the word untidy in her conversation with Miss Shaw. As she herself observed, she has also found "plenty of English and Scotch nurses untidy." But if Miss Shaw had not lost that button on the way to Brownlow Hill, the heated discussion on the question of national characteristics would not apparently have arisen.

### MORE NURSES WANTED IN DUBLIN.

The City of Dublin Nursing Institution, which was established 15 years ago, is in a prosperous condition. For the year just closed the receipts were £4,566, the largest sum yet reached. The number of nurses and probationers connected with the institution is 164, and yet, it is stated, the supply frequently falls short of the demand. In two years—we do not understand why the number of patients for each year, as well as the receipts, is not given—no fewer than 1,365 patients were treated, and it appears from the entries in the patients' remark book that the general treatment is of the most satisfactory kind.

### UNCERTIFICATED MIDWIVES.

Though many cases of suffering, and even of death, occur from the want of knowledge and timely skill on the part of women who, notwithstanding that they are uncertificated, practise as midwives, only a few come to notice. On the 13th inst., at Norwich, an inquest touching the death of a young married woman revealed another of the numerous sad results from lack of professional aid. We learn from the *Eastern Daily Press* that, at the suggestion of a juror, the midwife was censured, and the coroner said, "I also think it ought to go forth to the public that in cases where medical persons could not be afforded a certified woman can be obtained by applying to the lying-in charity. I don't think it is right, in the interests of the public in general, for women to follow an occupation of this kind without being properly certificated." Perhaps next session the registration of midwives may be rendered compulsory.

### THE SUTHERLAND BENEFIT NURSING ASSOCIATION.

The fourth annual report of the Sutherland Benefit Nursing Association is of a satisfactory character. According to the president, the Duchess of Sutherland, "the nurses, under the excellent supervision of Miss Stevenson, are being more and more appreciated in the county. Prejudices have been overcome, and gratitude gained." In the matter of fever nursing, the association co-operates with the County Council of Sutherland. The Council pay for a nurse for infectious diseases, and the Nursing Association, acting with the medical officer of health for the county, undertakes the responsibility of superintendence. This is an admirable arrangement, and seems to work well. In order that interest shall not be allowed to flag, a small library for nurses has just been started, and the nurses will undergo examinations in theory and practice to ensure thorough reading of the books.

### SHORT ITEMS.

Among the passengers who sailed for South Africa last week on the "Dunottar Castle" was Miss Lucy H. Williams, who goes to fill the post of matron at the hospital, Port Elizabeth. Miss Williams trained at Addenbrooke's, Cambridge, and has held various appointments, her last post being sister midwife at Queen Charlotte's Hospital. Her many friends and fellow-workers will wish her every success in her new appointment.—A course of eight lectures by Miss Margaret Sewell, warden of the Women's University Settlement, Southwark, on "Forethought in Charitable Work," has been arranged by the Joint Committee of the Women's University Settlement, the National Union of Women Workers, and the Charity Organisation Society, to take place at the Westminster Town Hall on consecutive Fridays at half-past eleven a.m., beginning on Friday this week. Owing to illness Miss Sewell will unfortunately be unable to deliver the lectures in person; but Miss Octavia Hill has kindly undertaken the task of reading them.—The result of an inquiry on the part of a Special Sub-Committee of the Govan Parish Council as to the adequacy of the nursing staff, is that they have recommended that one certificated nurse and one probationer be appointed to each of the 12 wards of the hospital, and that there be one nurse to supervise and one senior probationer in each ward for night duty, making a staff of one lady superintendent, one night superintendent, 12 certificated nurses, and 28 probationers. These recommendations have not yet been adopted by the council.—It was mentioned at the annual meeting of the Banbridge District Nursing Society that Lord and Lady Arthur Hill had collected £39 towards the fund. Lord Arthur Hill, who moved the adoption of the report, and other speakers referred in the warmest terms to the work done by Miss Carew since her appointment as nurse.—At St. Jude's, Peckham, on Tuesday evening, St. Luke's Eve, a special service was held for hospital nurses, of whom a number attended in uniform. Father Hopkins was the preacher.

### Lectures to Surgical Nurses.

By H. A. LATIMER, M.D. (Dunelm), M.R.C.S. of Eng., L.S.A. of London, Consulting Surgeon, Swansea Hospital; President of the Swansea Medical Society; Lecturer and Examiner of the St. John Ambulance Association, &c.

### RICKETS—ITS NATURE AND TREATMENT.

*Continued from page 24.*

### INFLAMMATIONS OF BONE.

As the result of the rickety changes in the long bones of the limbs they also become bent, curved, and twisted. These changes are particularly marked in the limbs of the lower extremity of the body, for here the weight of the body has to be supported, and it is only natural that in their softened state the bones should yield. So it happens that the various deformities known as "knock-knee" and "bow leg" are met with, and the tibiæ and femora become curved from front to back, as well as laterally. It is for the remedying of these deformities that the surgeon is called in.

When the softening process has been going on for a while, if the child improves in health and the disease is arrested, the bones become "ossified," and when this hardening change has been effected the distorted limbs are left in their twisted and deformed state. It is now that the surgeon steps in to remedy, by operations, the deformities which have occurred. It is true that when the bones are in a soft and plastic state he is sometimes called in with a view to correcting the distortions which are taking place, and that he is often successful in doing so by applying suitable splints to the limbs and by seeing that the child is placed in the most favourable condition for the recovery of his health by being supplied with suitable food and medicines, whilst breathing fresh air, and living in a place bathed in sunlight. But he often does not see the little sufferers in time to put them under this treatment, and is only applied to when the mischief has been done and deformities resulting from it have to be remedied. For the rectification of the curved bones he performs the various operations of "osteotomy," which consist in making incisions at suitable spots into the soft parts of the limbs and down to the bones which are to be dealt with, dividing these bones with chisels, breaking them to complete their separation, and then re-setting them in a proper line; finally the limbs so operated upon are placed in plaster-of-Paris, or other suitable splints, and the broken bones are left to be re-united in their new and correctly adjusted positions just as they are if fractured accidentally.

These operations of osteotomy are most successful in their results, as a rule, and have been performed with increasing frequency on that account, as the advantages which follow their performance have become more widely known. If performed with due antiseptic precautions, the wounds made by them heal in the kindest manner, and although the surgeon is, practically, making a compound fracture when he operates, no untoward result follows from his interference. I have operated over and over again in cases of rickets with the happiest results, and have restored limbs which have been hitherto curved and distorted to their normal straightness and correctness of outline. As nurses, you will have little to do in your attendances upon such patients, for the wounds which have been inflicted during the operations do not require any subsequent dressings, as they are dressed once and for all on the operating-table, and are covered by firm material, which is left *in situ* during the process of cure. Your duties thenceforth consist only of the ordinary ones of an attendance upon an invalid.

Other diseases affecting bones to which I shall call your attention are osteitis or inflammation, caries or ulceration, and necrosis or death, of them. Mr. Walsham has pointed out in the clearest manner in his work on "Surgery" that if you would understand the processes involved in inflammation of bone you must regard them from the point of view of the soft parts connected with them. These soft parts of a bone are the membrane, or skin, which encloses them as a sheath on the outside, and which is called the periosteum; a membrane lining the canal hollowed out of its interior, in which the marrow lies, called the endosteum; and a prolongation of this latter into the little spaces forming the intricate structure of the bone itself. Inflammation may occur in any one of these parts. If it should take place in the outer membrane we call the resulting process periostitis; if in the inner membrane, endostitis; if in the bone itself, osteitis. The phenomena of the inflammation here differ in no way from those of other inflammations as I have described them to you in a former lecture. The same features of pain, heat, redness, and swelling occur here as elsewhere, and the same results of resolution, thickening, suppuration, ulceration, and mortification may take place. I will not describe the various symptoms of these complaints to you, as these lectures do not call for detailed descriptions of disease as they would do were they addressed to surgeons. I will content myself with telling you that the pain from which the subjects of them suffer is oftentimes of a severe character, as might well be expected when one considers that it is occurring in rigid and unyielding structures, and that it is mostly aggravated at night time, and that there is a peculiar liability to pyæmia when the deeper-seated parts of the bones are inflamed, owing to the fact that the rigid nature of their structure keeps parts open, and allows of the entrance of septic materials into the veins. So, great care has to be exercised during operations upon bones to guard against the entrance of micro-organisms, or for the destruction of them by antiseptic agents should they have lodged there.

When a bone is breaking down, in particles, by a process analogous to that of ulceration in soft tissues, it is said to be carious. To hasten the process of separation of decayed bone and to cure the disease by removing dying tissue from its neighbourhood to healthy parts (for by mere contact diseased parts in the body will contaminate adjacent sound ones) the affected part is cut down upon, when carious, and all the softening and softened parts of it are removed by the gouge; the hole left after this operation is gently filled with some antiseptic gauze, which is removed and replaced daily, or by a drainage tube, for the wound can only satisfactorily heal by the process of granulation, and must be kept open until this new material has grown up from its bottom to the surface.

The process of gangrene in bone is known as necrosis It is peculiar, in that the dead bone becomes enclosed in new bone, through which one or more holes exist. The soft parts covering this diseased bone are inflamed and present channels, or sinuses, through which pus oozes. The dead part is called a sequestrum, and is finally separated from the living bone surrounding and enclosing it by ulceration; it then lies loosely imprisoned, and when the surgeon deems that the proper time has arrived for him to remove it he does so by cutting down upon the part which is affected, enlarging one or more of the openings with cutting forceps, and then extracting it. As in the case of caries, the part is now plugged or drained until the wound has healed.

You will often have to attend to these cases, for a great many people seek admission to hospitals for the cure of diseased bones. If you are called upon to do the necessary dressings for them, be gentle in your manipulation of the parts, and thoroughly antiseptic with your hands and instruments; gentle, because harm is done to the delicate new tissue by roughly stuffing dressings into it, and pain also is caused; and cleanly, because of the extra liability which exists for septicæmic or pyæmic processes to be started there.

### South Africa as a Field for English Nurses.

JOHANNESBURG, BULUWAYO, EAST LONDON.

At this particular juncture South Africa will probably take a prominent position in a nurse's calculations as an opening for her energies. Therefore, it will be of interest to our readers to contrast the advantages and disadvantages of such a field. The following details are given us by a correspondent of experience :—

Starting with the Transvaal, there is little doubt that Johannesburg, in its normal condition, offers, perhaps more than any city in the world, scope for the nurse's skill and energy. This statement must be taken with caution, however, in the sense of meaning a nurse's Eldorado, for the great mining metropolis boasts a hospital of its own, and a staff of trained nurses which rarely requires augmenting from home. At Cape Town and elsewhere in our South African colony plenty of nurses train, and, their certificates gained, their minds turn readily to a career in the golden city. An English nurse would require considerable influence to be a likely candidate for a vacant post; and she should only seek it if she is willing to brave all—agreeable or disagreeable—which is connected with the name of a nurse in a Johannesburg hospital.

Concerning private cases inthe city, these, I may venture to affirm, abound. To begin with, accidents are constantly occurring in the mines which literally girdle Johannesburg. Then, too, typhoid rages at all times and seasons. Bad water, and a system of drainage which is no system at all from a modern point of view, conspire to make this disease chronically epidemic. Remuneration, however, is a very open question for the nurse who is privately engaged. If her patient is the successful mining or business man, fees far exceeding any that are offered in England fall to her share; but if her case is that of the man whose lot is always to fail, even in the land of gold, the barest—if any—remuneration will be the reward of her labours. The former kind is to be found, in the main, amongst the Jews, who represent all that is most successful in Johannesburg. The unremunerative case is, unhappily, numerous, and is invariably the young Englishman, whose dreams of gold have known a bitter awakening. If luck comes his way in the future, he does not forget the nurse who has pulled him through, and rewards her in a manner more than proportionate to services rendered. Wives and children, too, of course come into the fever question, where the same inequalities of remuneration are to be found as with the fathers and husbands.

Consumption in all its stages makes the nurse in special request in Johannesburg. In earlier days the city established a legitimate reputation as a lung-healer—to the extent, indeed, of claiming to be the one spot in the world where life was possible with half the last remaining lung! But it has grown, and in the rush for wealth the sanitary needs of a large population have been overlooked. Refuse in all stages of decomposition, and innocent of disinfectants, is carted away daily to decay in the surrounding veldt. As the inevitable result of such a rough-and-ready process, the dry season finds clouds of germ-laden dust wafted back to the city, compelling patients who should be breathing fresh air freely, to sit with closed windows in their stifling rooms.

In Johannesburg, if anywhere, a nurse learns how to contend with everything which is unfavourable to her patients' recovery; for, in addition to this pernicious condition of the atmosphere, she has to put up with bad water, and frequently a scarcity of that. Moreover, most of the community live in "buildings"—flats in a certain sense, but lacking all the sanitary convenience of flats. House rent being at the rate of six guineas per month for a single room, a large proportion of her patients necessarily pass their days and nights sighing in vain for the breezes of an English common.

Up to the present, for lack of trained nurses, many of the poor invalids are dependent on the chance attendance of friends and acquaintances, moved to pity at their hopeless condition. Work, then clearly abounds for the professional nurse. Its remuneration alone is the uncertainty. Success, must, however, in the main come to the individual who knows her work and does her duty. With this in her mind, a nurse may feel that she might do worse than try her luck in this city of plenty and want.

In Pretoria the disparities of poverty and wealth are not defined so acutely. A hospital of some standing is, as at Johannesburg, supplied mainly with nurses from the Cape. Private cases are not so numerous as in Johannesburg, but on the whole remuneration is less of the up and down order of things.

There is little doubt that English nurses would find conditions more to their liking in Rhodesia than in the Transvaal. At Buluwayo both hospital and private cases can be had on lines of a more usual character; consequently the profession is less exposed to the variations of the financial barometer. The journey, if long, is straightforward, there being a rail route from the Cape. Rhodesia is a country of exceptional possibilities, and steering steadily for development, therefore it should be included by a nurse in her list of considered openings.

But most suitable, from an English point of view, is East London. A glance at a modern African map shows this really charming city to be in Cape Colony itself, on the coast, and about 150 miles north of Port Elizabeth. A steady trade makes its industry; and its money markets accordingly know nothing of the fluctuations inseparable from mining centres. Sick patients of all classes turn to the English-trained nurses, if available, in preference to those who have walked the colonial hospitals; and this applies not only to general nursing, but in particular to monthly nursing and midwifery.

### Death in Our Ranks.

Nurse Florence Cooper, whose remains were laid to rest at Bishopwearmouth Cemetery a few days ago, was the victim of typhoid fever contracted in Sunderland. Her duties led her into some of the worst parts of the town, and returning one night from nursing a case of typhoid she was heard to remark that if she took the disease she knew where she had got it. Unhappily, there were grounds for this expression, and ten days after the seizure by the malady Miss Cooper died, at the early age of 26. The sad incident shows it is not only in plague hospitals that nurses run the most terrible risks. Miss Cooper is the second nurse who has succumbed to typhoid fever this month. The other was Miss Kirkbride, whose last appointment was at Shooter's Hill, where, having nursed her patients successfully, she herself caught the disease, and died in a few days after she was attacked.

### To a Nurse.

By M. D. D.

I see you as I saw you those long weeks:
The prim white cap that hid the soft fine hair,
The spotless linen apron, and the hands
Trained to a nicety of skilful care.

I thank you for the silent sympathy
Told thro' the medium of expressive eyes;
The priceless boon of gentle touch that stilled
The weary aching of long agonies.

For sweet forbearance, and untiring skill,
I am your debtor thro' all time to be.
Lay those kind hands in mine, and say "Good-bye!"
I wish you good thro' all your destiny.

### The New Nurses' Home at Woolwich.

Eight years of steady work so endeared the Queen's nurses of the Woolwich, Plumstead, and Charlton Nursing Association to the inhabitants of the district that the most popular form of commemorating our Queen's Diamond Jubilee was considered the provision of an adequate residence for them. Wise heads as well as liberal hands were enlisted in the enterprise, and a fortnight ago the formal opening of 22, Nightingale Place, Woolwich, the result of sustained efforts, was announced in our columns. From Woolwich Station to the new home is a long steady climb. The locality improves in character as the summit of the hill is approached. Nearly at the top stand the handsome houses — commanding an extensive view — of Nightingale Place. The situation is an admirable one, for it is the centre of the large district administered by the association, which is a decided advantage to the nurses, because the hills around are too steep for much cycling to be possible. The air is delightful, whilst as a freehold property the home and the house adjoining—acquired as partial endowment—appear to afford excellent security for the money invested. The basement is only a foot or two below the ground level. It contains a large comfortable dining-room, convenient kitchens, and store-rooms. On the first floor is a lofty apartment, which makes a charming sitting room for the staff. This, like the rest of the house, is not yet fully furnished, but two or three easy chairs have been given by friends of the institution, and a Chesterfield couch has been promised. Behind is the room which was used as a dining-room by the former occupants; it is now the committee-room. It looks out over a long strip of garden, not large enough, unfortunately, for tennis or croquet, but quite sufficiently roomy for a lounge on summer afternoons. A third room on this floor, hitherto used as a butler's pantry, makes an excellent appliance-room. Here hang the nurses' cloaks, and here is a shelf for their bags, a cupboard for emergency loans, and another for liniments. Upstairs on the next floor is the nurses' bath-room, one large bedroom divided to make two comfortable small ones, and the superintendent's private rooms. Space did not permit of according to the latter the usual privilege of a private sitting-room as well as a bedroom, but this drawback is mitigated, to a certain extent, by giving her a spacious bed-sitting-room and the dressing-room adjoining. There is another floor, on which more bedrooms are arranged in much the same way as beneath. The household includes the superintendent—who was trained at Westminster Hospital, has been for six years one of the Queen's nurses, and has worked with the association for two and a half years—four nurses, and two maids.

### Novelties for Nurses.

### PERFUMES FOR THE BATH.

Those who are fond of perfumes will find Messrs. Quentin's perfumed wafers a pleasant addition to the bath. They are intended to soften the water also, and are supplied by J. A. Nones, 5, Bradford Avenue, E.C.

### "OKO" FOR BOOTS AND SHOES.

Many will be glad to know of "Oko," which is a preparation which helps to preserve the soles of boots and shoes, and also keeps the damp out. It is a liquid preparation, painted on with a brush, and only costs threepence per tin. It can be bought from Messrs. Keys, Hitch, and Co., 2, Talbot Court, Gracechurch Street, or through any bootmaker.

### Typical Patients.

### THE CRITICAL PATIENT.

Mrs. Giles was admitted to the medical ward of the C—— Hospital suffering from an attack of bronchitis. From the very first she showed a desire to know everything about the hospital and the nurses, and to criticise the management. Even the hospital cat (who looked consumptive) was not allowed to escape her notice.

"Well, I like your cheek!" Mrs. Giles said to him when she first saw him in the ward. "A gentleman like you a walkin' inter a room full o' ladies without so much as a blush —garn! You ain't worth your keep."

Mrs. Giles had strong faith in clinical thermometers. The first time the nurse placed one under her arm she remarked, when it was taken away, that it had done her "a power o' good. A sight better than dosin' yerself with Mother Seigel an' the like," she added.

When the visiting clergyman entered into conversation with Mrs. Giles he wished later on that he had only said "Good afternoon," for she had a way of tiring you out after the first 15 minutes.

"The on'y fault I 'ave to find with this plice is that there ain't no carpets. I was brought up on carpets, an' some'ow I don't get used to polished floors," she said to him, as he sat blushing under her gaze. (He was very nervous, and he longed to get away.)

"Yes," went on Mrs. Giles, "carpets is kind of 'ome like. 'Enry (my mate), 'e's just bin an' laid a nice one in our parlour. Oh! it's a fair beauty it is!"

The clergyman coughed. "You must not expect to have things as nice here as at home," he murmured.

"No fear," laughed Mrs. Giles, "you'd get your nose snapped hoff 'ere if you was to arst for a lightly done egg or a cup of fresh drawn tea of a mornin'. I ain't complinin' of this 'ere institution (she added hastily). If 'alf the women 'ere fancies their eggs done four minutes instead of free-and-a-'alf it on'y shows they don't know what's what."

"Are the nurses kind to you?" asked the clergyman.

Mrs. Giles laughed again. "Strike me dead! if they aint the queerest lot o' females I ever came acrost. Now, d'ye see that rum lookin' one with the carroty 'air, well, she carnt make me out at all. I guessed that as she keeps comin' an' puttin' a glass under my arm an' lookin' at it an' shikin' it. It did me a lot of good at first, but I don't feel any different now, so I reckon it's waste of time."

Mr. Palmer rose to go, but Mrs. Giles implored him to stay. She had something very important to say to him, and she would only keep him a minute. So he had to sit down very meekly again.

"D'ye see that young woman in the bed be'ind you, sir?" she almost whispered—"No, don't look just for a minute, 'cos she'll guess I'm talkin' about 'er."

Mr. Palmer waited the prescribed minute, then he fixed the person indicated with a stony stare.

"There! I told you so," whispered Mrs. Giles. "Aint she a reg'lar old cat—why she'd steal the very pillar under your e'd if she got the chanst."

"Indeed!" said Mr. Palmer.

"That ain't all," went on Mrs. Giles. "She paid me the shabbiest trick on Monday. She allays puts 'er air of a night inter six 'Indes curlers. Well, she 'eard me say on Sunday evenin' that I wished I'd brought my curlin' pins with me, 'cos a stright fringe don't suit me. Well, himagine my feelin's, sir, when I woke up on Monday mornin' an' took a look at No. 19. Why! believe me, she 'ad 'er 'air done up in twelve 'Indes curlers! An' when she saw me lookin' she winked as sarcy as you please."

### Across the Seas.

### A VISIT TO A LEPER ASYLUM IN INDIA.

Matunga is a small village lying about seven miles from Bombay. It contains a leper asylum for 300 patients, and on the occasion of my visit was quite full. The asylum was founded six years ago by Mr. Acworth, municipal commissioner of Bombay. Prior to the establishment of the asylum, the lepers prowled about the streets, friendless and homeless, spreading infection, and showing their terrible sores or maimed limbs to obtain the compassion and help of the public. On high days and holidays they were particularly *én evidence*, as the streets being crowded with holiday seekers, they were likely to earn quite a large sum of money. When the idea of a leper asylum was first started, the lepers raised a great outcry, and they were strongly supported by many of the public, who regarded them as public pets; however, in spite of protests the work of the asylum went steadily forward, and on a fixed day the police of Bombay pounced down on all the lepers they could lay hands on (there were about sixty to begin with), placed them in ambulances, and conveyed them to Matunga, where they were received by Dr. Chowksey. For some time the lepers sulked and refused to eat the good food prepared for them, or to take an interest in their surroundings, but gradually, by dint of kindness and patience, they saw that their good only was the first consideration of the authorities, and little by little they tried to do what they could to help also.

The asylum consists of several one-storied detached buildings situated in a beautifully-kept garden. The patients are arranged according to their castes, so that Christians, Mahommedans, high and low caste Hindoos, are kept quite separately. The wards are large, light, and airy, and are furnished with beds, tables, and chairs, and everything is very clean. The whole work of the asylum, excepting the compounding, washing, and cooking, is done by the lepers themselves, and for this they receive payment, according to the kind of work and the amount they are capable of doing. Very few of the lepers are bed-ridden; the majority were squatting in that peculiar attitude so typical of the native, and "doing nothing!"

On first entering a leper ward one is agreeably surprised that one does not see the terrible sights expected. The lepers all appear to be very much alike, and this, I think, is due to the leonine expression that they all have, for as the nose is largely affected in almost all cases from the beginning, the bone of the nose being twisted and nodulated, and in bad cases sinking and crumbling away altogether, causing the eyebrows to contract and meet, thus giving the leonine expression. Nearly all had lost a joint or so, and some even their limbs, but as, of course, all suppurating wounds are surgically dressed, one does not see the worst cases. The saddest sight is the school for leper children. When I visited it there were about twenty children of all ages being taught by a leper schoolmaster. Some were very much disfigured by the disease, but the others appeared to be all right, though I was told that they all had leprosy except one little boy, the child of leper parents, in whom the disease had not yet appeared, but who was there as his parents were unwilling to be separated from him, and it was regarded as certain that he would have it. Some of the children, from the loss of joints, were unable to walk or write; they appeared to be very cheerful, however, poor mites, and salaamed in a very ready fashion.

The little chapel for the Christians was prettily decorated with garlands of flowers and tinsel, and the harmonium was cleverly played by a leper boy. The Mahommedan mosque and Hindoo temple were full of devotees, and everything in perfect order. Outside each Hindoo ward was a "tulse" tree, growing on the top of a small pillar made of hardened earth and mortar, and gaudily painted. This tree is considered sacred by all Hindoos, and is tenderly nurtured. The greatest care is taken in cooking to please all creeds. For instance, rice is boiled in two ways. The Christians prefer each grain to be separate and fairly dry, whilst the Hindoos like their rice very sodden and served in a big round pudding shape. Hundreds of chappatties (pancakes made of flour and water) were being turned out, and every cooking vessel was beautifully clean. On feast days sweetmeats and luxuries are provided for all, and everything is done to brighten the lepers' lives and alleviate their sufferings. A remarkable illustration of the careful disinfection of soiled clothes is that the washerman, his wife, and children, who have been in the service of the asylum for ten years, are entirely free from leprosy. This appears to be little short of a miracle, as three years is the usual limit of immunity from infection.

### Appointments.

Hove Hospital for Infectious Diseases.—Miss Florence Hill has been appointed Matron. She was trained at King's College Hospital. Subsequently she has had charge of five senior wards and operating theatre at Jamsetjee Hospital, Bombay, and been sister of the abdominal ward of Sussex County Hospital, Brighton.

Royal Albert Edward Infirmary and Dispensary, Wigan.—Miss Jean McKerrow has been appointed Assistant Matron. She was trained at Charing Cross Hospital, and for the last twelve months she has been night superintendent at the hospital of which she has become assistant matron.

Hull Royal Infirmary.—Miss Mary L. Rannie has been appointed Lady Superintendent. She was trained at the Royal Infirmary, Edinburgh, and has since been senior night superintendent in the same institution.

Royal London Ophthalmic Hospital.—Miss G. M. Richards has been appointed Lady Superintendent. She was trained at the London Hospital, and has since been sister.

### Minor Appointments.

Chester Isolation Hospital.—Miss Fannie Dowson and Miss Florence Locke have been appointed Charge Nurses. Miss Dowson was trained at Chester General Infirmary, and Miss Locke at Bolton Infirmary and Dispensary. The latter has since been sister at St. Mark's Hospital, City Road, London, and also at the Royal Buckinghamshire Hospital, Aylesbury.

The Infirmary, East Dulwich Grove.—Miss Maude M. Harwood has been appointed Sister. She was trained at the Royal Hants County Hospital, Winchester, and has since been charge nurse at the Eye and Ear Hospital, Southampton.

Park Fever Hospital, Hither Green.—Miss Kate H. Todd has been appointed Charge Nurse. She was trained at the Royal Infirmary, Bristol, has since been engaged in private nursing in London and has been attached to the private nursing staff at Fitzroy House, W.

Bradford Union Hospital.—Miss F. A. Selby, Miss B. Dawson, and Miss L. Richford have been appointed Sisters. Miss Selby was trained at the Birmingham Infirmary, Miss Dawson at the Royal Southern, Liverpool, and Miss Richford at the Marylebone Infirmary, London.

### OUR CONVALESCENT FUND.

We acknowledge with pleasure the receipt of £1 from Miss Lucy L. and 5s. from "A. S. C." to the above fund.

### Nursing at the Greek Hospital, Alexandria.

By a Sister.

The Greek Hospital at Alexandria, is built in two blocks, connected above and below by verandahs, and stands in a large garden. The block on the right contains on the ground floor the ophthalmic ward with eye theatre, the out-patient department, dispensary, and laboratory. On the upper floor is the female surgical and medical ward, with rooms for first and second class patients, containing one or two beds, kitchen, a large empty ward, operating theatres, dressing and sterilising rooms. The block on the left contains on the ground floor the administrative offices, doctors' rooms, bed-rooms, linen-rooms, dining-rooms, and kitchen. On the upper floor are two wards, male surgical and medical, with their respective rooms for first and second class patients, kitchens, &c. The corridors are of coloured marble, which has an excellent appearance, and are wide, lofty, and very airy. The walls and ward floors are of cement, which cleans well, taking all disinfectants without staining. The wards contain twenty-six beds each, but as cases are never refused admittance beds are often put in the corridors.

There are five doctors, all Greeks, really clever men, introducing into their service every new invention for facilitating and raising the standard of the work, and for the benefit of the patients. The greatest cleanliness is insisted upon in every department. Most stringent antiseptic and sterilising measures are practised. One is often astonished to see how quickly the patients are ready to leave, most of the wounds healing by first intention. For the surgical dressings ordinary white gauze sterilised at a temperature of 124 deg. Centigrade is used, and at all operations only sterilised water temperature 134 deg. Centigrade is used for washing out the wounds. The preliminary preparations are made with "Savon glycerine" (a preparation of soft soap and glycerine), alcohol carbolic acide 2½ per cent., and sublime 1 in 1,000, after which all lotions are discarded. There is a bacteriological department; every doubtful disease is examined microscopically before the diagnosis is made.

The nurses are Greeks and natives, the sisters English; the former are under the sisters, who are supposed to train them. Both Greeks and natives are intelligent, and with kindness and firmness on the part of the sister will work well. In all difficulties the sisters are aided by their respective medical officers, and their work is made as easy for them as possible.

The language spoken is Greek, scarcely any English, although nearly all the doctors can speak it; so it is absolutely necessary for any sister contemplating nursing in the hospital to learn modern Greek. Everybody—doctors, nurses, and patients—is most kind in aiding one in this way, and it is soon picked up colloquially. The work is decidedly hard, but very good. No case, whatever it may be—surgical, medical, obstetric infectious—is refused admission. So the experience is excellent. It would strike an English eye as peculiar and somewhat droll to see the doctors and nurses (male, for in all the male wards there are male nurses) working in blouses; the former in holland, and the latter in striped or checked shirting. The sister's uniform is white, but at all operations sterilised blouses are worn by everyone present, including visitors.

The principal theatre is a large lofty room. The walls and ceiling are painted white. They are washed with a Vermorel machine after every operation, the disinfectants used being sublime 1 in 1,000, and after two hours a solution of carbonate of soda 1 in 100. The floor is of cement like the wards, with a drain in one corner to carry off all water. The table in ordinary use is an oval slab of thick glass on a painted iron stand, with a groove running round and across to carry off all water by a central hole. Two glass shelves, similarly supported, are on the walls, and a movable cupboard with glass shelves and door for some of the instruments. All water used for washing the hands, &c., is filtered through a Pasteur filter. An inner room is employed solely for the purpose of abdominal operations and vaginal hysterectomies, and is similar in every way to the large theatre. In one corner is a large cupboard of plate glass and nickel, with glass shelves, in which are kept most of the instruments; these are sterilised before and after using.

All infectious cases are nursed in a ward entirely separate from the main building, consisting of several rooms opening on to a verandah, and overlooking a pretty part of the garden, in which the convalescent infectious patients are allowed to walk. Hard by is a small church, used as a mortuary, and opposite is the post-mortem room.

There is a large laundry, where all the washing for the entire hospital is done.

The director has complete control of the establishment, and resides in the building, but the English sisters are responsible only to their respective doctors and the committee.

The hours off duty are uncertain, and depend upon the cases. Every sister has a bedroom to herself and a dining room in common. The food is peculiar to English taste, being cooked entirely in the Greek style, with oil in nearly every dish. Puddings, cakes, &c., one never sees, but the sisters are considered as much as possible, and fresh fruit is provided at every meal.

The patients are of all nationalities, and as a rule give no trouble whatever in the matter of discipline or cleanliness.

### Presentation.

Bute Hospital, Luton.—Miss Babcock, the matron at the Bute Hospital, Luton, has been presented on her retirement with a handsome brown leather dressing case by the Oddfellows and Foresters of the town; while the Committee of Management of the hospital have handed her a draft for the handsome sum of £59 in recognition of her labours during a period of more than nine years. On the occasion of the first presentation, a silver waist buckle was given to Nurse Ward, who is also leaving the hospital, as a mark of appreciation on the part of the members of the friendly societies. Miss Babcock, who was trained at St. Thomas's Hospital, will be much missed in the town.

Queen's Jubilee Institute, Edinburgh.—Miss Hadden, district superintendent, has been presented by the Queen's Nurses in Scotland with a gold watch and chain, as a mark of their affection and esteem, on the occasion of her leaving the Scottish branch to be Superintendent of the Central Home in Bloomsbury Square. The watch was engraved with her monogram and the inscription, "From the Queen's Nurses in Scotland. 1st October, 1899."

Bradford Eye and Ear Hospital.—Miss Duckitt, matron of the Eye and Ear Hospital, Bradford, has just been the recipient of a handsome silver-mounted dressing-bag and umbrella, presented by the committee, and a travelling bag given by the servants and porter, the happy occasion being her marriage.

### ROYAL UNITED HOSPITAL, BATH.

The following is the result of the examination for medals and certificates at this Hospital: Nurse Blanche Howell gold medal; Nurse Isabella Wardle, silver medal; Nurses Anna Lawrence, Katherine Roberts, and Alice Hayward certificates.

### Echoes from the Outside World.

AN OPEN LETTER TO A HOSPITAL NURSE.

At the time I write there is practically no important definite news about the war in South Africa. If there has been a big battle the information has not yet reached us, owing partly to the wires having been cut. Meanwhile, the interest is divided between Mafeking and Kimberley, the former because it is believed that Colonel Baden-Powell's forces are smaller than those of his antagonists; and the latter because of the presence of Mr. Cecil Rhodes, who, with his customary hopefulness, declares that Kimberley is "as safe as Piccadilly." This is in spite of the rumour that £5,000 has been offered for him by the Boers, "alive or dead." In London we have had two excitements this week—the big meeting in the City, at which Sir John Lubbock, speaking in support of a resolution of confidence in the Government, said that Mr. Chamberlain "had combined the meekness of Moses with the patience of Job"; and the opening of Parliament, which was necessary in order to obtain the money indispensable for carrying on the war. Of course, the Government have the support of the bulk of the Opposition, though the session may last for two or three weeks, and the financial proposals of the Chancellor of the Exchequer excite a certain amount of reasonable criticism. But nearly everybody seems to think that Lord Salisbury and his colleagues, having done their best to keep the peace, are now doing their best to bring the war to a speedy and successful end.

Surely the latest action on the part of the Queen must do something towards convincing the French people that there is still some good left in us, in spite of all the horrid things which the Parisian papers have of late been saying. Most of us have quite forgotten the "Leda" incident. A French smack, "Etoile de la Mer," fishing within the three-mile limit, off Dungeness, last summer, was chased by H.M.S. "Leda," and a shot fired struck a Frenchman named Loth with a fatal result. Whether under the circumstances no compensation could be claimed from our Government I do not know, but her Majesty, out of her private purse, has just forwarded to the British Vice-Consul at Boulogne 10,000 francs (£400), which on Monday was handed over to the father of the young steersman who was unfortunate enough to lose his life. The kind action was rendered the more valuable because it was accompanied by an expression of regret for the incident, and sympathy with the family of the deceased sailor.

Another little bit of news with regard to the Queen which will interest you is that recently there has been automobile practice in the Royal Mews at Windsor. Autocars of some description are now so frequently met upon the high roads that it has been thought desirable to accustom the royal horses to the latest invention, and thus prevent any chance of an untoward accident alarming, if not injuring, any members of the Royal Family. Accordingly for some little time a skilful automobilist has been testing the nerves of the horses in the Windsor stables, first causing them to walk round the vehicle whilst stationary, then making the steeds stand still whilst the autocar drove around them, and, lastly, letting the horses move freely about whilst the machine did all it could to alarm them. The consequence of this strange drill is that in future the coachmen will have no fear of the possible bad behaviour of their horses even, should they meet a perfect procession of motor cars.

The "luck" has been against Sir Thomas Lipton, for though the "Shamrock" was fairly beaten by the "Columbia" on Monday, the race on Tuesday was in favour of Mr. Iselin's yacht simply because the topmast of the challenger's vessel snapped off short, and she had to retire from the contest. The "Shamrock" must now win the rest of the three races, or the coveted cup will remain on the other side of the Atlantic. We all wish Sir Thomas success—for yacht races, like cricket matches, are never lost till they are won—and if ever a man deserved it, he does, for his good temper has been as unfailing as his pluck. In any case, it will be a consolation to him to know that his sportsmanlike spirit is immensely appreciated, not only in his own country, but also in the United States. It speaks volumes for his kindness of heart—does it not?—that when the "Erin" got alongside the "Shamrock" on Tuesday, after the accident, his first exclamation was one of relief that nobody was hurt.

Although hitherto, notwithstanding all that has been written about the cruelty of taking the osprey's feathers, fashionable ladies still continue to allow them to adorn their hats, the army has been more considerate. An appeal was made to the War Office representing that the osprey plumes worn by officers in their hats were only obtained after a great sacrifice of bird life, and an order has been issued that for the future the osprey plumes are to be replaced in every case by ostrich feathers. The colours, with a few exceptions, are to be the same, and the stems carrying the plumes will fit into specially designed sockets. It is inconceivable that women, whose hearts are supposed to be more tender than those of men, will continue to permit themselves, for a caprice, to indulge in such needless cruelty as robbing thousands of young egrets of life when even officials, who are supposed to have no feelings, have declined to be participators in the offence.

Does it not seem strange that whilst farmers in England find it so difficult to make both ends meet there should be room for the importation of 330 tons of New Zealand butter, which will arrive here some time next month? That there is a demand for the colonial article is abundantly proved by the fact that the quantity sent over the last two years has this year been exactly doubled, a venture which would not have been undertaken if the exporters in New Zealand had not been sure of their market. The remembrance of these statistics made me feel quite sad as I roamed about the British Dairy Show at the Agricultural Hall. All the arrangements for the successful carrying on of dairy work seemed so complete, the numerous cows so comfortably stalled, the fresh and tempting pats of butter, the noble cheeses from various parts of the provinces, together with the handsome sides of bacon—all these intensified one's regret that it is impossible for our farmers to turn out dairy produce in sufficiently large quantities to prevent our needing so much from elsewhere. Two new features of special interest this year are the milkers' competition and the contest for the lightest and best cakes and scones, made with skim milk. The butter-making tournaments are always much appreciated by the public. On Tuesday we had to wait our turn to get a chair to watch them. Some of the dairymaids were so bonny looking, and with their rosy cheeks and bright faces were by far the most picturesque part of the show. Had it not been for the strains of the band of the Coldstream Guards, I could have believed I was down in the depths of the country rather than in Upper Street, Islington.

### Hospital Sketches.

### THE LITTLE PANTOMIME GIRL.

"Gertrude Allverton, aged thirteen, of Greek Street, Soho," was the entry on the admission-sheet, dated Friday, November 2nd. She was a well-developed and strong little girl for one of the class to which she evidently belonged, but the vehicle that had run over her as she was merrily dancing to the sounds of a piano-organ at the corner of a busy little thoroughfare off Oxford Street had injured her cruelly. She was unconscious, and a cursory examination showed that several of her ribs were broken. An elder girl who had witnessed the mishap and who knew her well, had accompanied her and the constable in the shabby four-wheeler to the Royal Central West Hospital, where they arrived at five in the afternoon. A heavy fog had hung about all day, and at four a cold drizzling rain had lent its unwelcome aid to the general depression. All the gas jets in the Casualty Receiving Room were full on and the doors were kept closed as far as practicable, and yet the fog within was not dispelled.

From the elder girl it was ascertained that Gertrude was one of a family of two whose mother died a year ago. The other child was a boy of nine, who at the time of the accident would be at school—if not playing truant. The father was a badly-paid potman at a public-house in Tottenham Court Road, and was, Sundays, Christmas Days, and Good Fridays excepted, engaged in the bar the whole year round from very early morning till midnight. He had his meals at the public-house, and only went to the two rooms he knew as home to sleep and to pet his children.

At six o'clock the father arrived at the hospital. A neighbour had informed him of Gerty's accident, and he had begged his employer's manager to spare him an hour in order that he might go and see for himself the extent of his child's injuries. He was distressed to find that they were so serious, but was comforted by the assurances of the doctors, who told him that once located in the ward the little sufferer would receive the best and kindest treatment.

Gerty regained consciousness at midnight, and was bewildered on finding herself in a hospital ward. Her first action was to burst out crying, and then to refuse to tell the night nurse whether it was because of any acute pain. She sobbed for a long time and alarmed nurse, who warned her that she would make herself weaker still, and that if she did not cease she must send for the doctor. The fear of a visit from the doctor at once frightened her into silence, and she became communicative and told nurse that she was not crying from pain, but because, since her mother's death the previous year, she had acted as housekeeper to her little brother Billy and her father, who would "'ave to go orf at six in the mornin' to his work without 'is cup of 'ot tea, wot he had had reg'lar" ever since she had held her responsible post; and she added, with hesitation, there was also another reason why she wanted to be at home, but that that she could not tell nurse.

Towards daybreak she fell off into a peaceful sleep, and was not awakened till noon, when a member of the visiting-staff saw her, and deemed it advisable to perform a further slight operation.

At tea-time she came to a good deal, and inquired anxiously for her father and Billy. The Sister thereupon dispatched a note to the father, bidding him visit his daughter the following Sunday afternoon if possible, and enclosing him a visiting card available for two. On hearing of this Gerty revived considerably, and, smiling happily, dropped off to sleep again.

On Sunday morning the house surgeon, who saw her, told her that she was "doing fine," and promised her, in reply to the pressure of her eager pleading, liberty in a few weeks. The knowledge that she was not to be kept in hospital for ever and ever appeared to cheer her wonderfully. Why she so greatly disliked being kept from her bare home and the streets, especially at such a season of the year, the Sister could not imagine, but the news that she would be free again in a few weeks' time made her almost cry out for sheer joy.

Nearly all the children who come to the Royal Central West say that they regret leaving; it may strike some hospital-dreading reader as odd that it should be so, but it is nevertheless a fact, and one of which we, at the hospital, are very proud. And at Christmas time, when visitors in goodly numbers are allowed, the little ones who have had an opportunity of tasting the sweets of freedom pay us a visit and express themselves as not only ready, but yearning, to be back. Numbers of them, you see, are not homed and loved as they might be—more's the pity.

Two o'clock struck, and the visitors, who in many cases prefer to come an hour too soon and wait in the street, presented their cards at the front entrances, and swarmed all over the interior of the buildings. Husbands welcomed wives, wives welcomed husbands; fathers and mothers, sons and daughters, brothers and sisters, grandparents and grandchildren, welcomed each other in a way that only patients in a hospital for the very poor sick can do. But to little Gerty, lying there at death's door, no one came. The first quarter of an hour passed in seeing new faces and in looking on at hearty greetings; the second quarter she spent in wild conjectures as to her father's and Billy's lateness; the third in dismay at being forsaken by the only two from whom she had really expected love and comfort; and the last quarter of the weary hour she buried her face under the bedclothes and cried as she thought of the happiness about her as compared with her own desolate wretchedness. Three o'clock struck, and she waited still. Presently she began to think that they could not be coming at all. The time allowed for visitors ended at four. Then she suddenly opened her eyes and put down the sheets, and there before her stood the father with Billy at his side all in a tremble, but with a new sailor cap, which he held shoulder high that she and all who cared might see the bold gold lettering upon it. She was too overcome to speak to them, but father leaned over and kissed her, and Billy was so nervous and awkward that instead of meeting her lips with his he knocked her white forehead with his round, hard, and highly-polished chin.

The father explained that he would have arrived at two o'clock had it not entailed his getting leave from the manager again, a step he was extremely loath to take, for potmen are quickly discharged if they upset the great manager's pleasure or plans in any small way. And Gerty recollected then that the father was on duty till three on Sundays, and again from six till eleven, so that the working man may not be deprived of his dinner and supper beer.

"Arst the Sister if I shall be able to get better in time for the pantomimes, will yer, Father?" was her first remark on gaining composure. Even Billy's new hat had failed to put her off the one all-important question, the question that had made her uneasy from the moment she regained her senses. So the father timidly put the query direct to Sister, who then grasped the situation and understood why it was that her little charge had been so much the victim of unrest and so utterly impatient of confinement in hospital. Of course, the pantomimes would soon be in full swing, and already the girls and boys of proper age who lived within a wide radius of the Lyceum and Drury Lane and other theatres were all agog to be one of the engaged for the gorgeous representations. Gerty had been living for months in feverish anticipation of the glory and triumph that were to be hers when she appeared on the real first night in all the splendour and animation of the magnificent spectacle. The occasion had been talked over by her and Polly Stubbins, and Emma Brown, and Maria Thompson, and Betsy Carpenter hundreds of times, and at the very moment she was run over she was with these playmates practising a step that she and they had heard was likely to be in demand. Old Mrs. Hunter, who was known as a sort of local agent in Soho for the various stage-managers, had received Gerty's application for a job very favourably, and if all went well there was no doubt about the question of her appearance on the boards. Gerty was admitted by the girls to be one of the most blithe and graceful dancers in Soho, and truly dreadful would be her disappointment if at this, the eleventh hour, she should be prevented from making one of the excited throng.

"Oh, yes, we'll have you well in time," were Sister's answering words. And never were words heard with deeper gratitude. No prisoner listened with more gladdened ear to a repeal of their sentence than did the small child of Soho to the promise of the hospital Sister that she should have her liberty restored to her in time for rehearsals for the Christmas pantomimes.

Content with the news received, Gerty had Billy to sit on the side of her bed, and father on the stool at her head. They talked incessantly until at four o'clock the bell rang out clear and unmistakable as an indication to all and sundry visitors that the time of privilege was over. It was hurriedly arranged as they kissed good-bye that Billy should have a half-holiday on the Wednesday afternoon, and that he and Jessie Wilkinson, the elder girl who witnessed Gerty's accident, should come as visitors, and that on Sunday next father should again come soon after three with Billy.

The patient's condition improved rapidly, and by Wednesday the colour was beginning to make signs of reappearance in her cheeks. Her diet had been changed somewhat, and her spirits at the thought of seeing Billy and Jessie Wilkinson were higher than the Sister cared for.

"You must be a good, quiet girl, and not be so restless," she said, "or we shall have you thrown back again."

So when Billy and Jessie arrived she tried to be demure and calm; but, oh, it was so very difficult. Poor Billy was much neglected; he had perforce to forlornly amuse himself with his new sailor hat, which Gerty, as housekeeper, had told him he ought not to have put on, as it was much too good to wear on week days. Gerty and Jessie talked on and on about the pantomime, and Jessie promised to call upon Mrs. Hunter and deliver a long message from Gerty to the effect that she was not so very seriously hurt after all, and that the sister-in-charge said she would be quite well enough to take part in the rehearsals.

"Be sure and tell 'er as she ain't to cross orf my nime because she's 'eard as I've come into the 'orspital, and tell 'er that there ain't a gel in Soho as will be more reg'ler in coming for 'er drills ner me," were the parting words as Jessie and Billy, with the other visitors, left the ward.

But, alas and alas! Gerty was not to take part in the pantomime. On the Friday—the day when, as theatrical folk have it, the ghost does not walk—her little life ebbed silently away. The fractured ribs were followed by complications, and the little dancing girl, and the embryo housekeeper, was at rest.

### To Nurses.

In order to increase and vary the interest in the *Mirror*, we invite contributions from any of our readers in the form of either an article, a paragraph, or information, and will pay a minimum of 5s. for each contribution. All rejected manuscripts are returned in due course, and all payments for manuscripts used are made at the beginning of each quarter, i.e., January 1st, April 1st, July 1st, and October 1st.

### Everybody's Opinion.

**[Correspondence on all subjects is invited, but we cannot in any way be responsible for the opinions expressed by our correspondents. No communication can be entertained if the name and address of the correspondent is not given, as a guarantee of good faith but not necessarily for publication, or unless one side of the paper only is written on.]**

### DEATH OF A CHILD IN KENSINGTON INFIRMARY.

"Bee" writes: Is it right that a ward, and especially a children's ward, should be left at all? This is a great fault in most of our hospitals. The staff is not sufficient for the requirements, consequently the ward must be left at times, as the nurse on duty has so many calls on her time and her work is often scattered. Yet she is responsible. In the present case it appears the nurse took every precaution, yet the accident occurred. The manner of fastening was certainly not wise. Would it not have been better to have used the usual straps across the chest with armholes, thus fastening the child to the bed?

### WORKHOUSE NURSING.

"Once a Workhouse Nurse" writes: What a pity "E. R. W." spoilt such a pleasing account of workhouse nursing by a few remarks at the end of her letter. First, I do not see that a nurse sacrifices her professional standing by nursing in a workhouse. I know of two army sisters who gave up their posts to nurse in a workhouse, and they did not feel that they had sacrificed their positions in the nursing world at all; in fact, they were inclined to think the other way. In regard to the illiterate guardians, that is a trial, and requires the tact and patience of a lady. Therefore, if ladies in every sense of the word would take up workhouse nursing I think our workhouse infirmaries would indeed improve. But it would not be even just to wish to turn out those nurses who have tried to do their best for the sick poor, though they are not ladies. They have undertaken what so-called ladies have so far shrunk from doing.

### ONE OF THE FAMILY.

"Bee" writes: I quite agree with "Morganwg" that so much depends on the nurse herself as to her comfort and the manner in which she is treated by the members of the household as to where she takes her meals, &c. If a nurse conducts herself as she should and takes pleasure in her work, instead of making everything a trouble, she, in return, will generally receive courtesy and respect from all around her. On the other hand, if the nurse is used to "kitchen company," she is naturally most at home there; but the poor patient must suffer in such cases. I am sorry to add that occasionally, when working with another nurse, I have been surprised to see the upset in the house, the "airs and graces" and want of thought on the part of the nurse, where self is always first and the suffering of the patient only a secondary consideration; but these nurses are, I am sure, in the minority, for they are a disgrace to their profession.

### NURSING IN FEVER HOSPITALS.

"Sister V." writes: I have read with much interest the letters on the training given in fever hospitals. Having been charge nurse just lately for over a year at one of the hospitals under the Metropolitan Asylums Board, I can speak from experience as to the advantage of special training in that branch of nursing after general work. It is true in my own case, as well as in the case of other charge nurses I know, that before entering a fever hospital, after three or four years' training, I had never given a nasal feed, nor even seen one given. Of that fact I am not ashamed, not having been so fortunate as to have had the chance. No doubt some assistant nurses would think it rather clever to be able to teach the charge nurse in that respect, but myself I should prefer to inform the doctor of my ignorance, and request him to let me see him give it. With reference to what "Credenda" says about some charge nurses taking two hours for dressing, it is hard to believe. I cannot see how it could be managed out of the few morning hours, which are sometimes too short considering the work that has to be done. Where I was we never got more than twenty minutes for dressing, and were bound to be back in that time, in order to let the first assistants go. We also did all the treatment, as well as taking temperatures, and I feel sure that when we sat down, and did "next to nothing," we did not feel any the less our sense of responsibility. Of course, if anything went wrong, the charge nurse, and not the assistants, would have to take the blame.

"A Londoner in the North" writes: It seems to me that "Credenda," in her letter, is airing her own feelings about certain charge nurses through the medium of your valuable paper. Her assertions are very sweeping and absurd in the extreme, and it is strange that she has remained so long under a Board which employs charge nurses whom she accuses of neglecting their duties wholesale. It is ridiculous to suppose that a charge nurse considers her duties finished after having taken temperatures. It is true that an assistant nurse may have to show a charge nurse how to give a nasal feed, for it is not a common operation, and with 12 years' experience I have only seen it done once. We certainly cannot respect a woman who takes two hours each morning to "dress," but happily such are few and far between. I think "Credenda" might have said that one out of every ten in only a figure-head. In this hospital we charge nurses do the principal of the treatment when on duty, and much of the menial work also; and if any little extra work arises other than that which comes in the regular routine, the assistants, or most of them, look to the charges to perform it, and we do a hundred and one things of which the assistants never know or will know as long as they take so little interest in their work.

"A Private Nurse" writes: A great deal has been said regarding nursing in the Metropolitan Asylums Board Hospitals. Will you allow me a little space to say a few words about my experience in one of them? I spent more than a year in one of the largest as a charge nurse, and I can safely say that the nursing staff is a most efficient one. One or two of the charge nurses were promoted from assistants, but the greater number are trained nurses. It is absurd to say that some charge nurses consider their work done when they have "taken the temperatures." It would, indeed, be a waste of money to give a salary of £36 to charge nurses who would do no more, but anyone who knows the working of a ward will understand that such a thing is impossible, and a little time would prove that their services could be dispensed with. Fever nursing, in a ward with twenty or more acute cases, is very interesting work to most nurses, and there are many things a charge nurse must learn which it is impossible to learn in a general hospital. Some of my assistants were splendid women and excellent nurses. In every branch of nursing, special or general, one meets with women who are not suited for the work, and will never be nurses, no matter what their training. While I held the post of charge nurse, I tried to teach my assistants as much as possible, and in most cases found them willing to learn. All right-thinking nurses will admit that a charge nurse's duty is not only to "see the work done," but also to help her assistants to do it. In the hospital to which I refer, lectures are given to the assistant nurses by the medical officers, and to the second assistant nurses by the matron. I was very sorry to give up my post, and only did so on account of my health.

"H. Green" writes: The suggestions by a charge nurse in a letter appearing recently in your columns is that one of the reasons for the resignation of the nurse is the impossibility of obtaining adequate help from assistant nurses, not trained. May I be allowed to state from my own experience, as well as that of others, that in the majority of cases this is not true. If the assistant nurses are not capable of giving adequate help, and are not equal to their work, why do some of the charge nurses gradually put the work and responsibility on to the assistant's shoulders? I have frequently seen a nurse sit in the ward practically doing nothing, and allow her assistant to do two nurses' work, and seldom, if ever, offer her any assistance. There are some who do not consider it their duty, but rather beneath their dignity, to attend to a patient's comfort if there is an assistant in the ward, even though her assistant may have so much work to do, in so short a time, that she is sometimes puzzled to know what to do for the quickest and best. Quiet remonstrance elicits the reply that a trained nurse is not engaged to work. Perhaps this is the reason why the officials sometimes prefer promoting their assistants; they must know by experience what they are capable of doing, and that they can rely upon them to give adequate help in the wards when required. These hospitals are supported by the public, principally the working class, and we do not suppose that they are desirous of supporting nurses unwilling to work, whether they are trained or not, when they themselves have to work hard for little money. If the nurses were to be more loyal to the officials and show a little more consideration to their assistants, no doubt there would be fewer changes. Certainly there are exceptions; nurses who are womanly women, who not only consider their patients, but also their assistants, and who remember their own days of probation. These are the nurses who win respect, and are of great assistance to the officials by helping to keep the staff. We are looking forward to the days when the Metropolitan Asylums Board hospitals will become recognised training schools, and when the assistants will be no longer considered inferior to the average probationer.

### IS THE NURSING PROFESSION OVERCROWDED?

"Lily" writes: I observe in your columns that a matron of a large and important hospital recently remarked, "Whatever becomes of all the nurses trained in small hospitals, I cannot think!" Then there is more utter nonsense about the necessity of small hospital nurses being "drawn from a different class" from her sisters of the same profession. Now, why on earth should this be so? I have heard doctors say that nurses in small hospitals (where there is no clinical school) get a much better training, as they have greater opportunity of gaining practical knowledge where there are no students to do the work which the doctors have not time for. Also at operations the small, hospital nurse has a better chance of seeing and hearing all that is done and said than if she had rows of students in front of her. Of course, everyone has guessed by this time that I have been trained in a small hospital myself, and so I have, in one with about an average of 50 patients. There were about fourteen nurses on the staff, both for hospital duty and private work. We had our medical and surgical wards and lectures (two each week) on anatomy, physiology, and practical nursing, and an examination at the end of each term. It is more the training that makes the good efficient nurse than the number of cases she has seen in the wards. I didn't think there was a matron living who didn't know that. Not taking fever cases (except enteric), we were each marched off for some months to "a large and important" fever hospital for training in that branch, and we certainly had our eyes opened; we were not used to the kind of nursing that was carried on there, and now that I think of it the nurses certainly were "drawn from a different class" to ours; they cared nothing for the comfort of their patients; they did not tell the truth, and didn't expect others to do so either. Mrs. So-and-So was ordered one pint of cream daily; it was generally left till it became sour, and was thrown out, or found its way into someone's tea. You were sneered at if you made the patient's beds in the evenings as well as in the mornings. There was a case of a poor woman who had a bedsore; I have seen the nurses drag the drawsheet from under that poor thing, causing her intense agony, instead of gently raising her from side to side and taking it out properly, as we should have done in the "small hospital." None of us who have finished our three years' training has had the least difficulty in getting appointments; indeed, in some cases they were offered to us before we had time to look for them. I have been connected with this institution for the last seven or eight years, and I have never yet known a nurse to be "out of work" for more than a month. Some are in England and some in Ireland. I was once a patient in a "large and important hospital," and the treatment I got was anything but what it should have been; very different to that which nurses or patients received in that despised "small hospital." Although the latter was poor, we had our up-to-date steriliser, &c., before some larger hospitals thought of such things.

### For Reading to the Sick.

ST. LUKE'S DAY.

LUKE, the beloved physician.—*Col.* iv. 14.

Be not slow to visit the sick, for that shall make thee beloved.—*Ecclus.* vii. 35.

The world's a room of sickness, where each
Heart knows its anguish and unrest;
The truest wisdom, then, the noblest art,
Is his who skills of comfort best;
Whom by the softest step and gentlest tone
Enfeebled spirits own,
And love to raise the languid eye,
When, like an angel's wing, they feel him fleeting by.
Feel only—for in silence gently gliding,
Fain would he shun both ear and sight;
Twixt prayer and watchful love his heart dividing;
A nursing father day and night. —*Keble.*

**Reading.**

There are no nobler professions than the two devoted to the guiding of the soul, and the healing of the sick. To those who recognise their high calling, who live in daily realisation of its sacredness, how blessed, how dignified, is the lowliest act of service performed for the Master's sake. "In that you did it unto the least of these My brethren, ye did it unto Me."

When our physical frame, mysterious, complex blending of soul and spirit, is wracked by acute suffering; when, perhaps, for the first time we realise the awful capacity for feeling, the dread self-isolation, the dumb, paralysing power of pain; when, in these supreme moments our best and dearest are powerless to help us, then we turn to those ministering angels, the skilled physician, the gentle, capable nurse, for services which only those trained in the school of suffering can render efficiently. Then through the lingering night watches, whose silence vibrates with the pulsations of suffering, how eagerly the wearied eyes and strained ears wait and watch for the hour that brings the presence of those who come with healing in their voice and touch. Be gentle, be brave, be true, those of you who are called to exercise your sacred offices! For at no time can the influence of example be more strongly felt than when, helpless, and too often hopeless, some suffering soul comes under your care and turns to you for consolation. It is then, by your presence, that you can arouse the dormant qualities of fortitude and patience in the heart of this stricken soul astray in life's maze. May it, in His good time, when the probation of suffering is passed, be spared to go out into the world once more, no longer nerveless and self-distrustful, but strong with the faith inspired by the contemplation of services unflinchingly rendered, in their smallest detail, with loving exactitude, and thus thank God for the hour when He called it aside to suffer and to learn.—*F. B.*

Hark, hark! a voice amid the quiet intense!
It is thy Duty waiting thee without—
Rise from thy knees in hope, the half of doubt—
A hand doth pull thee—It is Providence!
Open thy door straightway and get thee hence;
Go forth into the tumult and the shout!
Work! love! with workers, lovers all about!
Of noise alone is born the inward sense
Of silence; and from Action springs alone
The inward knowledge of true love and faith.
—*Mac Donald.*

### Notes and Queries.

The contents of the Editor's Letter-box have now reached such unwieldy proportions that it has become necessary to establish a hard and fast rule regarding Answers to Correspondents. In future, all questions requiring replies will continue to be answered in this column without any fee. If an answer is required by letter, a fee of half-a-crown must be enclosed with the note containing the enquiry. We are always pleased to help our numerous correspondents to the fullest extent, and we can trust them to sympathise in the overwhelming amount of writing which makes the new rules a necessity.

Every communication must be accompanied by the writer's name and address, otherwise it will receive no attention.

*Abroad.*

(31) Will you kindly tell me if there is any prospect of work for a nurse who wishes to go abroad, either in New Zealand or Nassau, West Indies; or if there is any demand for women workers in any occupation? If so, to whom could I apply for information as to voyage, &c.?—*H. S.*

New Zealand has more trained nurses than she wants. You can find out exactly where women workers are wanted and their prospects from the Emigrants' Information Office, 31, The Broadway, Westminster.

*Army Nurse.*

(32) I am a fully-certificated nurse, and am very anxious to get into the army, and more so to go out with troops to the war. Is it necessary that I should be an army nurse before being sent out, and can you tell me of any society that will send me out?—*Nurse G.*

So far, only the sisters of the Army Nursing Service are being sent out with our troops. Their places in home hospitals are being filled with the sisters of the Army Nursing Reserve Service. You must belong to one or other of these services in order to be eligible for nursing service with the army.

*Puerperal Fever.*

(33) Will you kindly tell me, as I have assisted in nursing a puerperal fever case, how long a time must I have finished with the patient before I am justified in nursing another patient in maternity?—*Anxious.*

It is not a matter of time so much as of cleanliness and antiseptics. If it were possible to restore a septic nurse to a condition of asepticity in the same way as a septic instrument—such, for example, as a scalpel—is dealt with there would be no reason why, as soon as the process was over, she should not go to another case. But it is not possible to do so, and as the outcome of much experience it seems to be generally accepted that a month should intervene between leaving a case of puerperal fever and attending another confinement. It must be clearly understood, however, that lapse of time has but little effect in diminishing septicity. It is the utilisation of that time so as to ensure repeated baths, repeated washings of the hair, the free use of antiseptics to the hands, and the thorough sterilisation of the clothing by washing and boiling that renders the nurse again fit to go to another case. If a nurse, after having a bad case, were to pack up her working clothes, and her syringes, her scissors, and all the rest of her working paraphernalia in her box and go off for a holiday, and if, on her return, she were to step into her "things" just as she had left them, she would be almost as septic and dangerous a person at the end of the month as at the beginning.

*Combined Training.*

(34) Could you kindly inform me if there are any hospitals which would give a three years' certificate after one year's training? I have already had two years' training in a clinical hospital, and am prepared to give up the salary for the year.—*Nurse Sarah.*

You must first find out if the Local Board of Ireland will recognise the training that you already have if supplemented by a further course of study. Write for this to the secretary in Dublin. If they will, your next step is to select a good hospital which awards certificates for one or two years, and then to write a second time to the Secretary of the Local Government Board asking if the combined teaching of the two schools will be recognised as qualifying you for the post of superintendent nurse. This approved, try and arrange with your second school to give you a certificate declaring that you have had three years' training; that is, two years in such and such a hospital and a further period in their own. Keep all documents and letters.

*School Girls.*

(35) Can you recommend me a book on nursing suitable for lecturing to school girls, giving all useful hints for home nursing?—*Sister.*

No one should lecture on a subject in regard to which she does not possess knowledge; but if the book is wanted merely to suggest the form and sequence of the proposed lecture, either the little handbook on nursing issued by the St. John Ambulance Society or Humphrey's Handbook of Nursing or the Lewis' Nursing would serve the purpose. Probably the first-mentioned would serve as well as any.

*St. John Ambulance.*

(36) 1. Can you tell me if there are any ambulance (St. John) classes held in York? 2. Also, can you tell me of any recognised training hospital where they receive probationers at 20 years of age?—*Inquirer.*

1. Write to the Secretary, St. John Ambulance Association, St. John's Gate, Clerkenwell, E.C., for particulars. 2. "The Nursing Profession: How and Where to Train," published by the Scientific Press, will give you a list of hospitals receiving probationers at the age you mention.

*Paid Probationers.*

(37) B. R. wants to know at what hospitals in London probationers for training where a small salary is given are received; and when is it most convenient to apply?

Applications are received by most hospitals at any time, but the accepted candidate has to wait for a vacancy before she can enter. See "The Nursing Profession: How and Where to Train," price 2s., from the Scientific Press, W., for regulations and salaries.

*Colonial Nursing Association.*

(38) Will you kindly tell me the address of the Colonial Nursing Association?—*H. W.*

Imperial Institute, South Kensington, S.W.

### Travel Notes.

By Our Travelling Correspondent.

### XXXVIII.—BRUSSELS.

Those amongst you who are hoping to take a short holiday now or about Christmas will do wisely to think of some bright town, such as Brussels, or Antwerp, or Paris, for it is hardly the season for strictly rural rambles. After our long dry summer we cannot expect an equally fine autumn, so that towns where one can study picture galleries, churches, and even shops in shelter, should be our aim. Let us take Brussels to-day under our consideration. It is a bright and charming town with all the gaiety and cleanliness of a lesser Paris. Trams, omnibuses, and railways abound, and there are innumerable excursions to be made weather permitting, whereas if rain is persistent there is plenty to be seen within the town itself.

### Expense of the Journey.

This is but trifling—first-class return for thirty days, £2 8s. 6d. ; second-class return, £1 10s. 11d., or if you would like to manage with stricter economy, and will go from Tower Bridge viâ Ostend in one of the General Steam Company's boats, you may do it first-class return £1 5s., second-class return £1 0s. 1d. I have been this way and found it very agreeable. These boats, however, only sail twice a week, on Wednesdays and Saturdays. The length of the voyage need not frighten you, because several hours are spent in the Thames before coming to the open sea. The boats are very good. I made the passage one night in a pretty considerable storm, and there seemed but little rolling considering the weather, which prostrated most of the travellers.

### A Week in Brussels for Five Pounds.

Some few years since I wrote a series of articles entitled "A Week on the Continent for Five Pounds," first experimenting myself to be sure that expenses could be kept within this limited amount. Brussels was one of the places of call. I triumphantly remained ten days for the five pounds, being the proud possessor of fivepence when I landed in England. I am an old hand at travelling, and I do not suppose that the inexperienced will be able to manage that, but a week may be reckoned on with care. I need hardly warn you that you must not start for the Continent with five pounds only in your pocket. That would be very dangerous ; you should at least have from three to five in reserve in case of emergency, for it is a serious thing to find oneself penniless in a strange land.

### The Sights of Brussels.

Foremost one naturally places the Cathedral and the Grande Place. Ste. Gudule is situated on the sharp slope of a hill, of which there are several in Brussels. Go in by the west door, so that you may approach by the imposing flight of steps which lead up from the street. One of the great beauties of the church is the magnificent stained glass of the windows, much of it dating from the thirteenth century. That in the chapel of Notre Dame de la Deliverance, on the south side, is three centuries later and decidly inferior, but the general effect is good, giving the interior of the church that dim richness so peculiarly pleasing, and that we miss so much in the magnificent cathedral of Antwerp. The high altar is entirely level with the floor, in no wise raised. I like to stand in front of it, in the subdued light of the gloaming, and return in fancy to the cruel night when the decapitated body of the gallant Egmont rested here awaiting burial. He and Count Hoorne were executed in the Grande Place in front of the Hotel de Ville by order of Alva. Egmont was the idol of the people, and his dead body was visited by crowds, but the sterner Hoorne, lacking Egmont's popularity, was alone in death as he had been in life, and his body was huddled away out of sight of all and buried obscurely.

### The Grande Place.

This is probably the most interesting square in Europe for its size ; the exquisite Hotel de Ville is the finest in Belgium, with the exception of that of Louvain. Immediately opposite stands the Maison du Roi, in an upper corner of which Counts Egmont and Hoorne passed the night previous to their execution ; at right angles stand the guild houses. That of the butchers shows a swan, the skippers mount the stern of a vessel as their emblem, and all are richly carved and gilded. On the south-east is a large building that used to be the public weigh-house, now occupied by municipal offices. The execution of the counts took place between the Hotel de Ville and the Maison du Roi, and the tradition is handed down that they reached the block by traversing a scaffolding fixed from it to their room, so that there should be no possibility of a rescue from the infuriated crowd below. The beautiful statue erected to their memory originally stood on the spot where the execution took place, but, on account of traffic, it was moved some years ago to the Place du Petit Sablon. Immediately behind the Hotel de Ville is the celebrated Mannikin fountain, to our English minds an extremely vulgar piece of work, but greatly beloved by the inhabitants. On all great fête days he is dressed in gala costume.

### The Place Royale.

The Place Royale and the Place des Palais are both full of splendid buildings, as their names suggest, but be content to look at them from the outside. Fees are charged for an interior view (in the case of the Palais Royal 2 frs.), and really the money is wasted ; these palatial residences, with their little toy park adjoining, are not in any way interesting.

---

### TRAVEL NOTES AND QUERIES.

Rules in Regard to Correspondence for this Section.—All questioners must use a pseudonym for publication, but the communication must also bear the writer's own name and address as well, which will be regarded as confidential. All such communications to be addressed "Travel Editor, 'Nursing Mirror,' 28, Southampton Street, Strand." No charge will be made for inserting and answering questions in the inquiry column, and all will be answered in rotation as space permits. If an answer by letter is required, a stamped and addressed envelope must be enclosed, together with 2s. 6d., which fee will be devoted to the objects of the "Hospital Convalescent Fund." Any inquiries reaching the office after Monday cannot be answered in "The Mirror," of the current week.

St. Moritz (Nurse).—I fear there is nothing at all at your terms. It is a place divided in two. At the Baths of St. Moritz, where the Kurhaus is, the very lowest would be 8 frs. per day ; at the village one mile and a quarter distant the lowest terms would also be 8 frs. There are one or two pensions ; the prices I do not know, but where hotels are dear the pensions are also so. I will give you their names if you wish. Ask your doctor if he cannot advise another place in Switzerland not so expensive. Davos Platz is much more reasonable, so is Mürren, but probably these places would not suit you so well. There is an English church at St. Moritz, and you must be warmly clothed, taking nothing but woollen clothes of various thicknesses. Let me hear again what you decide, and I shall be pleased to help you if I can.

Florence or Rome (Perplexed).—There is much to be said for both in your case. If your friend is greatly interested in art galleries, archæology, and in ecclesiastical architecture she would find all these in Rome, and agreeable society also. If she cares much for scenery and enjoys walks and expeditions, I think it possible she might prefer Florence, for it has all the advantages of Rome, with what I may perhaps call easier surroundings. You need not be nervous about Roman fever. Avoid over-fatigue, and going into churches when heated. It is cold in both places in winter, but colder in Florence than in Rome.

Paris (Artist).—Yes, you can live as you propose comfortably for 6 frs. a day in many pensions in Paris ; there is a class of such houses which lay themselves out for the accommodation of artists of both sexes. (2) Not very much left ; old Paris has almost entirely disappeared ; in the quarter called the "Marais" some fine old houses still exist, and in the Faubourg St. Germain.

Brussels or Bruges (Claymore).—You can hardly compare the two. Brussels is like a very small Paris, whereas Bruges is quite a city of the past, quiet and sleepy. Living is cheaper in Bruges. Terms, 6 to 7 frs. Accommodation may be had in Brussels for that sum, but with less comfort. Second-class ticket to Brussels, 19s. 3d. ; Bruges, viâ Dover and Ostend, £1 1s. 6d. Viâ Steam Navigation Company's steamer from Tower Bridge to Ostend, first-class, 8s. 6d. You can also go to Brussels by this cheap route.

